# Tradeoffs between dispersal and reproduction at an invasion front of cane toads in tropical Australia

**DOI:** 10.1038/s41598-019-57391-x

**Published:** 2020-01-16

**Authors:** Crystal Kelehear, Richard Shine

**Affiliations:** 1 School of Life and Environmental Sciences, University of Sydney, Sydney, NSW 2006 Australia; 2 Department of Biology, Geology & Physical Sciences, Sul Ross State University, Alpine, Texas 79832 USA; 3 Department of Biological Sciences, Macquarie University, Sydney, NSW 2109 Australia

**Keywords:** Invasive species, Sexual selection

## Abstract

Individuals at the leading edge of a biological invasion experience novel evolutionary pressures on mating systems, due to low population densities coupled with tradeoffs between reproduction and dispersal. Our dissections of >1,200 field-collected cane toads (*Rhinella marina*) at a site in tropical Australia reveal rapid changes in morphological and reproductive traits over a three-year period after the invaders first arrived. As predicted, individuals with dispersal-enhancing traits (longer legs, narrower heads) had reduced reproductive investment (lower gonad mass). Post-invasion, the population was increasingly dominated by individuals with less dispersive phenotypes and a higher investment into reproduction (including, increased expression of sexually dimorphic traits in males). These rapid shifts in morphology and reproductive biology emphasise the impacts of the invasion process on multiple, interlinked aspects of organismal biology.

## Introduction

Any organism has a finite store of resources to allocate among competing demands such as maintenance, growth, and reproduction; and hence, natural selection is expected to fine-tune that allocation in ways that maximise lifetime reproductive success^1^. As a result, levels of investment into reproduction should depend upon competing priorities, with organisms decreasing reproductive output if investment into other functions yields higher benefits in fitness. Such tradeoffs may be especially clear during biological invasions, because of differences in evolutionary forces at the invasion front versus in long-colonised areas^2^. Individuals at an expanding range edge often exhibit unusually high rates of dispersal^3,4^, requiring energy that may decrease allocation of resources to other functions such as immune defence^5–7^ and reproduction^8–11^. The increased allocation to dispersal is exacerbated by the non-adaptive process of spatial sorting; individuals that allocate more energy into dispersal will likely be close to the range edge, even if more rapid dispersal does not enhance fitness^12^.

To test these ideas, we need to compare attributes of individuals at an expanding invasion front to conspecifics in longer-colonised areas. We can perform that comparison either through space (monitoring sites with different times since invasion) or through time (monitoring a single site as an invasion passes through). Most available evidence comes from spatial comparisons, which minimise confounding due to temporal (seasonal, annual) variation but introduce confounding factors that vary among sites (e.g. resource levels, predator abundance)^13^. Comparisons through time at a single site avoid those problems, but require longer-term monitoring and introduce confounds associated with temporally variable factors. In the current paper, we describe the results of studies over a three-year period at a single site, beginning soon after the initial arrival of a colonising species.

The invasion of cane toads (*Rhinella marina*, formerly *Bufo marinus*) through tropical Australia has attracted considerable research. Individuals in the vanguard disperse long distances every night, and exhibit phenotypic traits (such as longer legs and narrower heads) that facilitate sustained locomotion^14–19^. Spatial comparisons among toad populations suggest a tradeoff between dispersal rate and reproductive output: compared to conspecifics from long-colonised areas, females from invasion-front populations reproduce at low rates even under standardised conditions^20^, and males have smaller testes relative to body size^21^. We sampled toads at a single site through a three-year span to look for the same kinds of changes through time, and for predicted tradeoffs between dispersal and reproduction.

## Results

**Figure 1:**
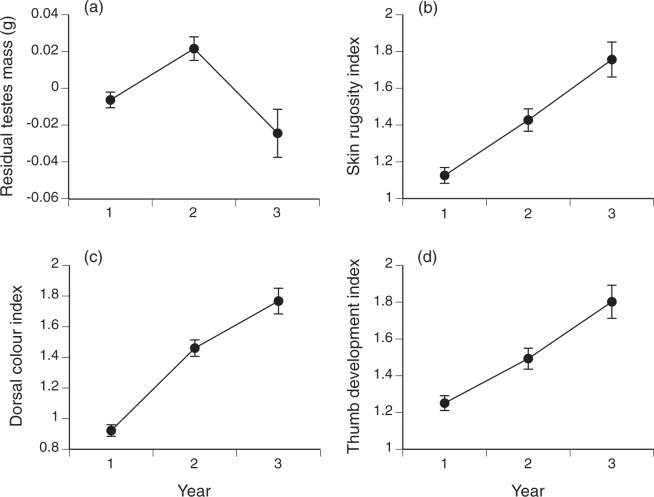
Variation in morphological traits of male cane toads (*Rhinella marina*) as a function of time since initial invasion.

Mean body sizes remained similar across the sampling period (Table 1), but there were significant shifts among years in several traits. In male toads, testes mass relative to body mass increased from Year 1 to Year 2 (significant with a posthoc test), then declined in Year 3 (ANOVA, *F*_2,655_ = 8.52, *P* < 0.0002; Fig. 1a). Changes in other sexually dimorphic traits were more straightforward, with mean values increasing through time for skin rugosity (*F*_2,718_ = 17.97, *P* < 0.0001; posthoc 1 < 2 < 3; Fig. 1b), skin colour (*F*_2,719_ = 61.11, *P* < 0.0001; posthoc 1 < 2 < 3; Fig. 1c) and development of the metatarsal tubercle (*F*_2,718_ = 21.82, *P* < 0.0001; posthoc 1 < 2 < 3; Fig. 1d). In females, ovary mass relative to body mass did not change significantly among years (*F*_2,424_ = 0.59, *P* = 0.56).

**Table 1:** Annual cane toad sample sizes and trait values.

Sample size	Year 1	Year 2	Year 3
	719	443	110
**Morphological traits**			
Snout-urostyle length (mm)	100.82 (0.48)	103.49 (0.62)	104.22 (1.24)
Body mass (g)	128.98 (2.19)	135.37 (2.79)	145.06 (5.63)
Head width (mm)	41.10 (0.20)	42.48 (0.26)	42.63 (0.52)
Tibia length (mm)	40.92 (0.19)	41.55 (0.25)	41.86 (0.49)
Sex ratio (% male)	60.22	60.95	78.90
**Reproductive traits**			
Total testes mass (g)	0.20 (0.006)	0.25 (0.009)	0.29 (0.019)
Total ovary mass (g)	5.89 (0.68)	7.53 (0.99)	7.91 (3.05)
**Rate of change (haldanes)**			
Relative head width (all)		0.23	−0.10
Relative head width (male)		0.27	−0.08
Relative head width (female)		0.18	−0.02
Relative tibia length (all)		−0.21	0.02
Relative tibia length (male)		−0.04	−0.05
Relative tibia length (female)		−0.49	−0.27
Relative testes mass		0.32	−0.55
Relative ovary mass		0.11	−0.14

Table shows mean values and associated SEs for data from each year of the study. “Relative” variables are residual scores from the linear regression of that trait on snout-urostyle length (linear traits) or body mass (mass-based traits).

**Figure 2:**
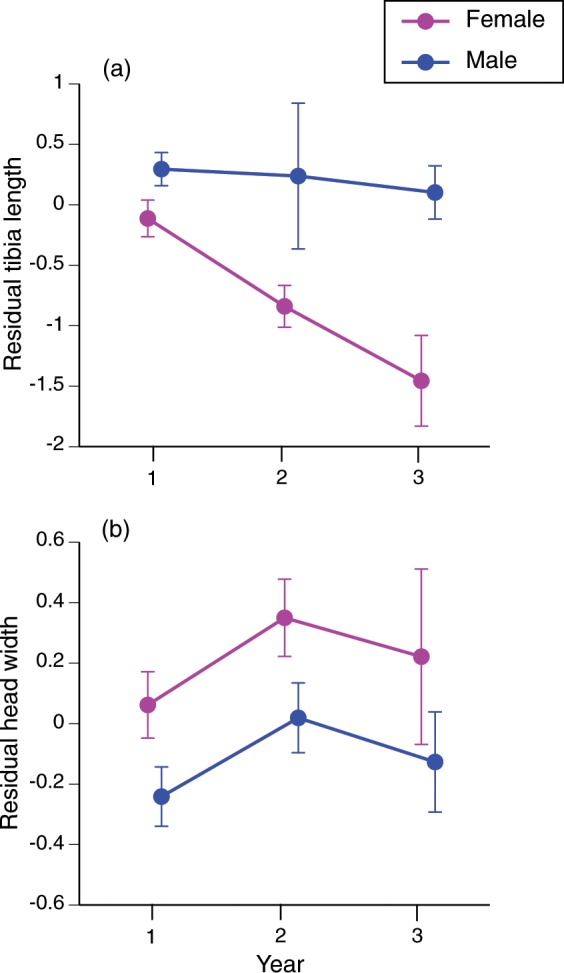
Sexual dimorphism in cane toads (*Rhinella marina*) as a function of time since initial invasion.

Changes through time were also evident for morphological traits associated with enhanced rates of dispersal, and known to differ between males and females^17,18^. Tibia lengths relative to body length declined rapidly post-invasion in female toads, but not males (interaction year*sex, *F*_2,718_ = 17.97, *P* < 0.0001; posthoc 1 < 2 < 3; *F*_2,1270_ = 7.92, *P* < 0.0005; Fig. 2a). In contrast, relative head widths increased from Year 1 to Year 2 in both sexes (main effect of year, *F*_2,1270_ = 6.72, *P* < 0.0015; sex effect, *F*_1,1270_ = 5.26, *P* < 0.022; interaction year*sex, *F*_2,1270_ = 0.08, *P* = 0.92; Fig. 2b).

Rates of change in quantitative traits can be expressed in Haldanes (one Haldane = a change of one standard deviation per generation^22^). Cane toads mature at approximately one year of age^23^, so annual rates of change equate to Haldanes. Rates of annual change in the traits that we measured ranged from −0.46 to +0.32 Haldanes between Years 1 and 2, and from −0.55 to −0.05 Haldanes between Years 2 and 3, with no obvious differences between sexes or between morphological versus reproductive traits (Table 1).

Lastly, variation in gonad mass relative to body mass among individuals was negatively (albeit weakly) associated with variation in relative tibia length in both sexes (males *n* = 656, *r*^2^ = 0.008, *P* < 0.025; females *n* = 425, *r*^2^ = 0.016, *P* < 0.01). Variation in relative head width was negatively associated with variation in relative gonad mass in females (*n* = 425, *r*^2^ = 0.022, *P* < 0.002) but not in males (*n* = 656, *r*^2^ = 0.0003, *P* = 0.68). If we include Year # as well as the morphological variables in an ANCOVA, the link between relative head width and relative gonad mass is strengthened in females (*F*_1,421_ = 10.31, *P* < 0.0015) whereas the same relationship in males remains non-significant (*F*_1,655_ = 0.01, *P* = 0.92). The same result is obtained with analyses including Year # as well as relative tibia length (tibia length effect for males, *F*_1,655_ = 2.90, *P* = 0.09; tibia length effect for females, *F*_1,421_ = 5.97, *P* < 0.015). These results imply that the negative correlation between variables related to dispersal versus reproduction in females was not a result of combining data for years with different mean values for each of these traits. Looking only at Year 1 (closest to the invasion front, and with the largest sample size), females with larger relative ovary masses had narrower heads (*F*_1,277_ = 13.32, *P* < 0.0003) and shorter legs (*F*_1,277_ = 9.89, *P* < 0.002).

## Discussion

When we first began sampling cane toads, a few years after they first arrived at our study site in tropical Australia, the population was dominated by individuals with dispersal-enhancing phenotypes (long legs, narrow heads^17–19^). Males at the invasion vanguard exhibited low gonad masses, and minimal development of sexually dimorphic traits. Over the next three years, those attributes shifted; newly-arriving toads showed phenotypic traits less strongly associated with dispersal (e.g., had shorter limbs), had larger testes (at least briefly), and displayed more highly-developed secondary sexual characteristics (e.g., skin rugosity). Within the overall sample, phenotypic traits associated with dispersal were negatively correlated with our measures of reproductive output. The reduction in tibia length post-colonisation was more rapid in female toads than in males, such that the degree of sexual dimorphism in this trait increased through time (Fig. 2a).

Although annual changes in relative ovary mass were not statistically significant, this result may tell us little because a female toad may have a small ovary either because she is non-reproductive or because she has recently spawned. Hence, ovary mass may not reflect reproductive rate. In contrast, testes masses may better reflect reproductive condition; and spatial comparisons have reported smaller testes in invasion-front toads than in range-core toads^21^. At a quantitative level, the rates of change in trait values per generation (in Haldanes) fell within the range reported in other studies of microevolutionary rates of characteristics under intense selection^22^.

In summary, concurrent changes in dispersal-relevant morphology and in reproductive investment accord well with predictions from theoretical models about post-invasion attenuation in dispersal capacity. Specifically, our data reveal the pattern expected from the predicted tradeoff between allocation of energy to dispersal versus to life-history functions^2^. Spatial sorting (accumulation of dispersal-enhancing phenotype at the expanding range edge) likely plays a role also, and the net phenotypic shifts through time likely reflect a combination of life-history tradeoffs and spatial sorting^2,12,15^. For males, the fitness disadvantages of lowered investment into reproduction may be minimal at the invasion front, where competitors are scarce; but those disadvantages rapidly increase post-invasion, with the rise in population densities (and hence, intensity of male-male competition). Operational sex ratios became more male-biased also (see Table 1), further increasing the intensity of male-male competition and hence, imposing increasing disadvantages to males with low investment into reproductive activities.

In general, our results from phenotypic changes through time are consistent with inferences based on comparisons through space. For example, a narrower head and longer legs have previously been reported in invasion-front cane toads^17,18^. Perhaps the most striking aspect of our results is the timescale of such changes. Spatial comparisons generally have compared populations with time-since-colonisation intervals at decadal or longer scales, whereas we saw rapid changes within a three-year period. Consistent with our results, temporal analyses reported similarly rapid shifts in energy balance^24^ and dispersal behaviour^15^ of toads at our study site post-invasion. Lacking data on attributes of offspring raised in common-garden conditions, we cannot distinguish whether these changes are driven by phenotypic plasticity or by adaptation.

One intriguing pattern in our data is the rapid change in sexual dimorphism in a morphological trait (relative limb length) that is highly correlated with dispersal rate and has shifted over the course of the toad invasion (longer legs at the invasion front^14,18^). Mathematical models predict that if one sex is intrinsically faster than the other, the fitness benefit to more rapid dispersal at the invasion front will be weaker in the more rapidly-dispersing sex (who otherwise will outpace the slower sex^25^). Male cane toads have longer legs than females, and hence are faster dispersers^18^. Thus, the disproportionate advantage to higher-than-usual rates of dispersal (=longer-than-usual legs) applies only to females at the invasion front. As soon as toads colonise an area, operational sex ratios no longer depend on sex differences in rate of dispersal. The advantage of unusually fast dispersal to females (relative to males) thus declines, resulting in a rapid decline in limb lengths in the slower sex (females); and hence, a shift in sexual dimorphism for this trait (see Fig. 2a).

Tradeoffs between dispersal ability and reproductive investment have been documented in many species, but involve a variety of proximate mechanisms. The simplest is for reduced reproductive investment to directly enhance dispersal rate: for example, lighter seeds can float further on the wind^26^ and female reptiles carrying a heavier clutch cannot run as quickly^27^. The tradeoff between flight and fecundity has been well-studied in insects^28,29^. However, tradeoffs are evident also at the genetic level: for example, artificial selection on fecundity generated rapid changes in wing morphology in crickets^8^. The tradeoff we have documented in cane toads also involves heritable traits (see above) and hence, is plausibly due to variation among individuals in allocation to competing functions. Individuals at the invasion front have evolved to disperse rapidly, via natural selection (access to abundant food at the front^24^), sexual selection (lowered male-male competition at the front^30^) and spatial sorting (non-adaptive winnowing of dispersal-enhancing genes across the invasion history^12^). These individuals exhibit morphological traits that enhance dispersal ability, with a commensurate decrease in investment into competing functions such as immune responses^6^ and reproduction^20,21^ (and current paper). As soon as the front passes, however, increasing population densities and attenuation of the advantages to dispersal may shift selective forces to favour phenotypes with less emphasis on dispersal relative to other functions^31^. Our data suggest that such transitions may occur very quickly, such that the distinctive phenotypes of individuals in the invasion vanguard are rapidly replaced by traits better-suited to the evolutionary forces at play after the system achieves spatial equilibrium.

## Methods

### Study site and species

We collected toads within Australia’s wet-dry tropics, centred on a site 60 km east of the city of Darwin (12°34′43.54″S, 131°18′51.55″E). Higher ground is dominated by savanna woodland, with extensive floodplains in low-lying areas. The area is hot year-round, with monsoonal rains from January to March in most years^24^.

Extensive research, based primarily on spatial sampling across the toad’s invaded range, has documented many differences between individuals from the invasion front versus range-core, and common-garden breeding experiments have confirmed that many of those phenotypic differences are heritable^6,19^. For example, the offspring of invasion-front individuals exhibit higher rates of dispersal, and more consistent directionality of movement^23,32^. Many of the evolved differences involve traits that enhance rates of dispersal; for example, invasion-front individuals have higher endurance^33^ across a wider range of abiotic conditions^34^, longer legs^14,18^, narrower heads^17^, and invest less energy into immune function^6,35^ and reproduction^20,21^. Invasion-front individuals also tend to be bolder and more active^36,37^. Some of the geographically divergent traits are highly heritable, whereas others are influenced by phenotypic plasticity also^19,23,38^.

### Sampling methods

Our sampling commenced in 2008, three years after toads first arrived^39^, and continued for three further years (see Supplementary Information). Population densities of toads increased each year during that period^40,41^, suggesting that the invasion front was still passing through the area and hence, the population should be subject to both selective tradeoffs and spatial sorting. Toad abundance then decreased in 2012 and thereafter^39^. We defined Year 1 as September 2008 to August 2009, Year 2 as September 2009 to August 2010, and Year 3 as October 2010 to June 2011. Thus, each year’s sample included the wet-season period during which toads actively disperse^42^. The abundance of toads was not significantly correlated with annual variation in precipitation over the period 2005 to 2012^39^, suggesting that our temporal comparisons were not strongly affected by variation in weather conditions. Extensive mark-recapture studies at our main study site detected no toads staying more than a single year; thus, all of the sampled animals likely arrived at their respective locations in the same year that they were collected. We hand-captured adult toads while they were active at night^43^. The toads were humanely killed and dissected the following day. We recorded body size (snout-urostyle length [ = SUL], body mass), head width, and length of the tibia. We removed gonads, blotted them dry, and weighed them to 0.001 g (Precision Balance FX-200i WP, A&D Company Limited, Tokyo, Japan). For males, we also scored the degree of development of sexually dimorphic traits on a 3-point scale for each of three variables; sexually active males develop more rugose skin, yellow dorsal colouration, and enlarged metatarsal tubercles on the thumbs^43^.

This work was approved by the University of Sydney Animal Ethics Committee (L04/1-2010/3/5193; L04/5-2010/2/5334), and all methods were performed in accordance with the relevant guidelines and regulations.

Data were checked for normality and variance homogeneity prior to analysis; no transformations were needed. Using JMP 13.0, we analysed temporal shifts in traits via ANOVA, using Year # (1, 2, 3) as a factor and phenotypic traits as dependent variables. Because testes mass increases with overall body mass, we used residual scores from the general linear regression of gonad mass on body mass as our measure of gonad size (calculated separately in the two sexes). To examine temporal changes in sexually dimorphic traits (relative tibia length and relative head width), we used Year # and sex as factors, and the morphological feature (residual score from the linear regression of that trait against SUL) as the dependent variable. To assess predicted tradeoffs, we calculated Pearson correlations between morphological and reproductive traits. To see if such correlations were confounded by annual changes in trait values, we also conducted ANCOVAs with year as factor, morphological trait (e.g. residual tibia length) as covariate, and relative gonad mass as the dependent variable. Non-significant interaction terms were deleted and main effects recalculated. We used Tukey post-hoc tests to locate significant differences revealed by ANOVAs and ANCOVAs.

## Supplementary information

Data for Kelehear and Shine.
